# The efficacy of pregabalin for pain control after thoracic surgery: a meta-analysis

**DOI:** 10.1186/s13019-023-02449-1

**Published:** 2024-01-03

**Authors:** Li Zhang, Hong Zhang

**Affiliations:** Anesthesia Department Operating Room, Chongqing Liangjiang New Area People’s Hospital, Chongqing, China

**Keywords:** Thoracic Surgery, Pain control, Pregabalin, Neuropathic pain

## Abstract

**Background:**

Pregabalin may have some potential in alleviating pain after thoracic surgery, and this meta-analysis aims to explore the impact of pregabalin on pain intensity for patients undergoing thoracic surgery.

**Methods:**

PubMed, EMbase, Web of science, EBSCO and Cochrane library databases were systematically searched, and we included randomized controlled trials (RCTs) assessing the effect of pregabalin on pain intensity after thoracic surgery.

**Results:**

Five RCTs were finally included in the meta-analysis. Overall, compared with control intervention for thoracic surgery, pregabalin was associated with significantly reduced pain scores at 0 h (mean difference [MD]=-0.70; 95% confidence interval [CI]=-1.10 to -0.30; P = 0.0005), pain scores at 24 h (MD=-0.47; 95% CI=-0.75 to -0.18; P = 0.001) and neuropathic pain (odd ratio [OR] = 0.24; 95% CI = 0.12 to 0.47; P < 0.0001), but demonstrated no obvious impact on the incidence of dizziness (OR = 1.07; 95% CI = 0.15 to 7.46; P = 0.95), headache (OR = 1.00; 95% CI = 0.30 to 3.35; P = 1.00) or nausea (OR = 1.24; 95% CI = 0.46 to 3.35; P = 0.68).

**Conclusions:**

Pregabalin may be effective to alleviate the pain after thoracic surgery.

**Supplementary Information:**

The online version contains supplementary material available at 10.1186/s13019-023-02449-1.

## Introduction

Thoracic surgery is recognized as one of the most painful surgeries [1–5]. These patients may suffer from obviously postoperative pain which may develop into chronic pain [6–8]. Postoperative pain after thoracic surgery is burning and stabbing, and it shares many features of neuropathic pain, because tissue damage causes hyperalgesia and allodynia due to the increased sensitization of dorsal horn neurons [9–12]. However, there are still lack of effective approaches to alleviate pain after thoracic surgery.

Pregabalin is one important drug to reduce the excitability of the dorsal horn neurons [13–15]. It was first introduced as one anticonvulsant and anxiolytic drug [16]. Interestingly, pregabalin has been successfully used to alleviate the neuropathic pain after knee, laparoscopic and spinal surgeries, and similar positive results are found for intercostal neuralgia in patients with post-thoracotomy pain [17, 18].

Several RCTs reported that pregabalin may have the capability to alleviate pain intensity after thoracic surgery, but the results were not well established [19–21]. Considering these inconsistent effects, we therefore conducted this meta-analysis to evaluate the effectiveness of pregabalin on postoperative pain for thoracic surgery.

## Materials and methods

### Study selection and data collection

This meta-analysis of previously studies did not need ethical approval or patient consent. It was conducted according to the Preferred Reporting Items for Systematic Reviews and Meta-analysis statement and Cochrane Handbook for Systematic Reviews of Interventions [22, 23].

We have searched PubMed, EMbase, Web of science, EBSCO, and the Cochrane library up to March 2023, using the search terms “thoracic surgery” AND “pregabalin”. The inclusion criteria were as follows: (1) study design was RCT; (2) patients underwent thoracic surgery; (3) intervention treatments were pregabalin versus control intervention. Patients were excluded if they had severe cardiovascular or respiratory diseases, impaired hepatic or renal function, and history of chronic use of analgesics.

### Quality assessment

The Jadad Scale was used to evaluate the methodological quality of individual RCT [24]. This scale consisted of three evaluation elements: randomization (0–2 points), blinding (0–2 points), dropouts and withdrawals (0–1 points). The score of Jadad Scale varied from 0 to 5 points. Jadad score ≤ 2 suggested low quality, while Jadad score ≥ 3 indicated high quality [25].

### Outcome measures

The following information was extracted: first author, publication year, sample size, age, male, smoking and methods of two groups. The primary outcomes were pain scores at 0 h and pain scores at 24 h. Secondary outcomes included neuropathic pain, dizziness, headache and nausea.

### Statistical analysis

A team consisting of three authors did the statistical analyses. Odd ratio (OR) with 95% confidence interval (CI) was applied to evaluate dichotomous outcomes, while mean difference (MD) with 95% CI was used to assess continuous outcomes. *I*^*2*^ statistic was applied to assess the heterogeneity, and significant heterogeneity was observed when *I*^*2*^ > 50% [26]. The random-effect model was used when encountering significant heterogeneity, and otherwise fixed-effect model was applied. We conducted the sensitivity analysis through detecting the influence of a single study on the overall estimate via omitting one study in turn or using the subgroup analysis. P ≤ 0.05 indicated statistical significance and Review Manager Version 5.3 was used in all statistical analyses.

## Results

### Literature search, study characteristics and quality assessment

**Fig. 1:**
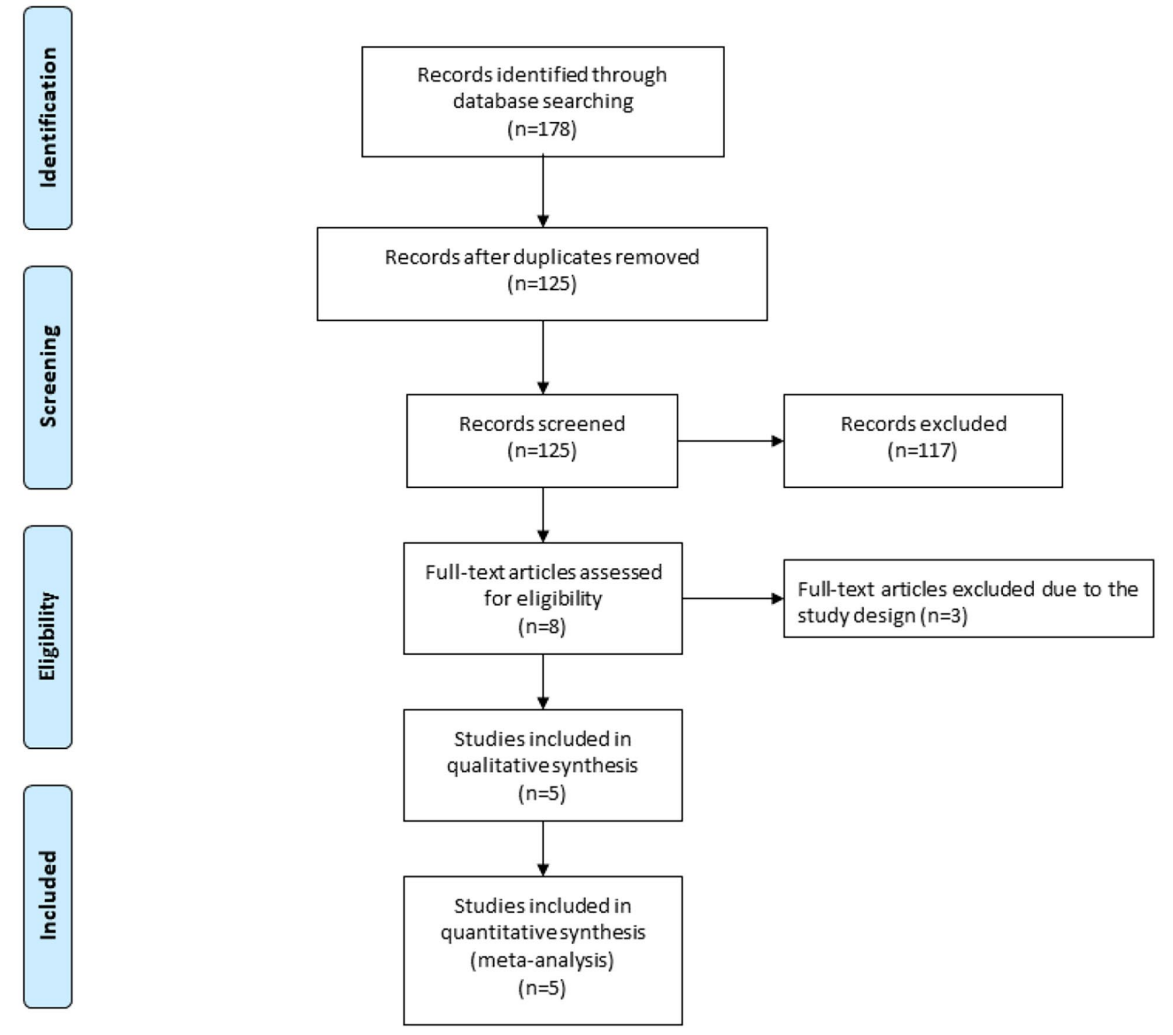
Flow diagram of study searching and selection process

Figure 1 showed the flow chart for the selection process and detailed identification. 178 publications were searched after the initial search of databases. 53 duplicates and 117 papers after checking the titles/abstracts were excluded. Three studies were removed because of the study design and five RCTs were ultimately included in the meta-analysis [19–21, 27, 28].

The baseline characteristics of five eligible RCTs were summarized in Table 1. The five studies were published between 2015 and 2019, and total sample size was 329. There were similar characteristics between pregabalin group and control group. Among the five RCTs, two studies reported pain scores at 0 h and pain scores at 24 h [20, 21], three studies reported neuropathic pain [19, 20, 28], three studies reported dizziness [20, 21, 28], two studies reported headache [20, 21] and three studies reported nausea [20, 21, 28]. Jadad scores of the five included studies ranged from 3 to 5, and all studies were considered to have high quality according to quality assessment.

**Table 1:** Characteristics of included studies

-	Author	Pregabalin group					Control group					Jada scores
		Number	Age (years)	Male (n)	Smoking (n)	Methods	Number	Age (years)	Male (n)	Smoking (n)	Methods	
1	Homma 2019	46	67, median	33	31	pregabalin (25 mg, twice daily)	46	70, median	28	25	non-steroidal anti-inflammatory drugs	3
2	Gaber 2019	30	46.9 ± 10.1	22	-	pregabalin 150 mg every 12 h	30	41.0 ± 14.5	21	-	placebo	4
3	Kim 2017	30	56 ± 12	13	-	pregabalin 150 mg 1 h before anesthesia	30	58 ± 9	17	-	placebo	5
4	Miyazaki 2016	33	66, median	15	-	pregabalin (75 mg, twice daily)	34	69, median	22	-	nothing	3
5	Yoshimura 2015	25	69.2 ± 9.0	12	-	pregabalin (75 mg, twice daily)	25	65.2 ± 10.4	12	-	nothing	3

### Primary outcomes: pain scores at 0 h and pain scores at 24 h

**Fig. 2:**
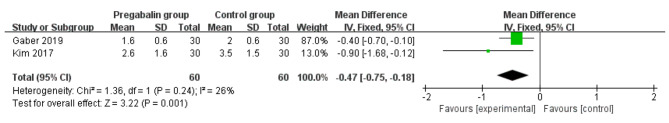
Forest plot for the meta-analysis of pain scores at 0 h

**Fig. 3:**
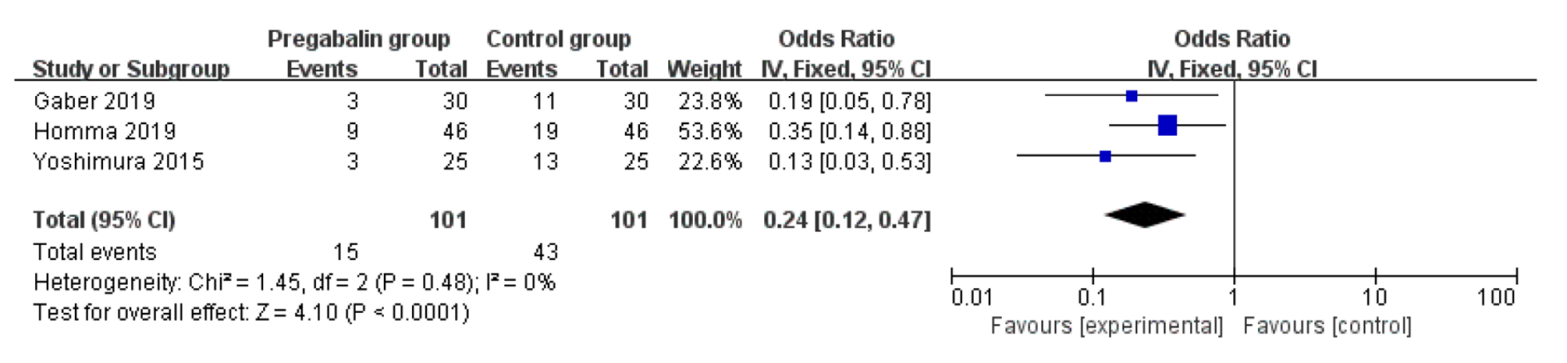
Forest plot for the meta-analysis of pain scores at 24 h

The acute pain within 24 h after the thoracic surgery was crucial for patients’ recovery and satisfaction. There was no significant heterogeneity remained for the primary outcomes, and thus the fixed-effect model was used. Compared to control group for thoracic surgery, pregabalin was associated with significantly lower pain scores at 0 h (MD=-0.70; 95% CI=-1.10 to -0.30; P = 0.0005) with low heterogeneity among the studies (I^2^ = 19%, heterogeneity P = 0.27, Fig. 2) and pain scores at 24 h (MD=-0.47; 95% CI=-0.75 to -0.18; P = 0.001) with low heterogeneity among the studies (I^2^ = 26%, heterogeneity P = 0.24, Fig. 3).

### Sensitivity analysis

There was low heterogeneity for the primary outcomes, and thus we did not perform the sensitivity analysis by omitting one study in turn.

### Secondary outcomes

**Fig. 4:**
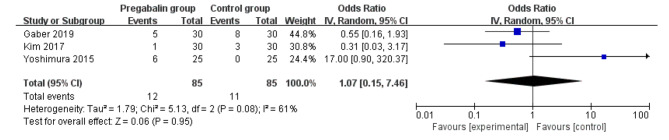
Forest plot for the meta-analysis of neuropathic pain

**Fig. 5:**
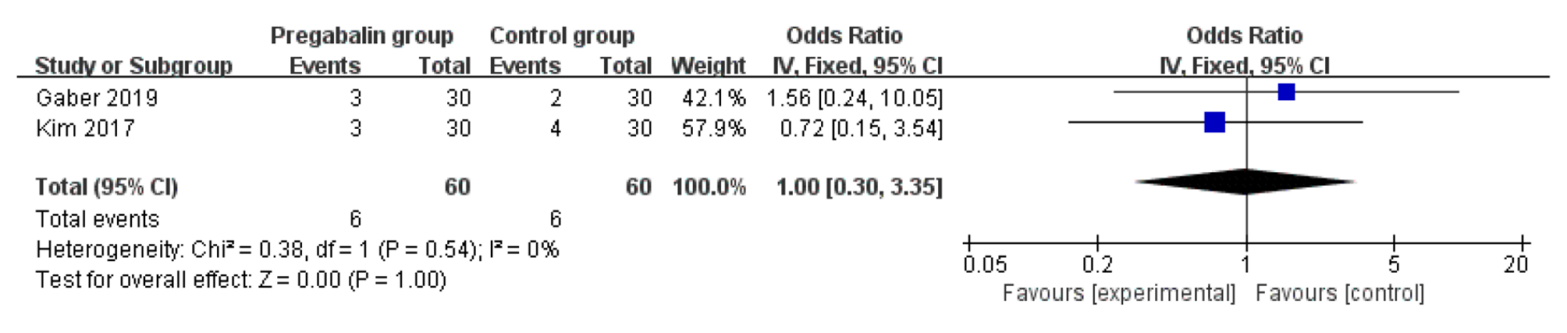
Forest plot for the meta-analysis of dizziness

**Fig. 6:**
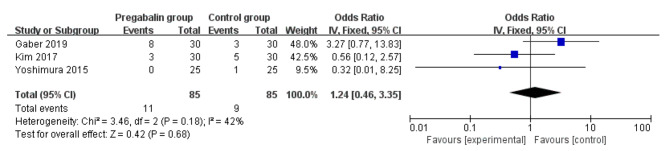
Forest plot for the meta-analysis of headache

**Fig. 7:**
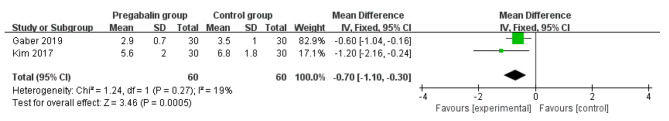
Forest plot for the meta-analysis of nausea

Pregabalin was also widely used to alleviate the neuropathic pain [13, 29, 30]. Compared with control intervention for thoracic surgery, pregabalin resulted in substantially reduced incidence of neuropathic pain (OR = 0.24; 95% CI = 0.12 to 0.47; P < 0.0001; Fig. 4), but showed no impact on dizziness (OR = 1.07; 95% CI = 0.15 to 7.46; P = 0.95; Fig. 5), headache (OR = 1.00; 95% CI = 0.30 to 3.35; P = 1.00; Fig. 6) or nausea (OR = 1.24; 95% CI = 0.46 to 3.35; P = 0.68; Fig. 7).

## Discussion

Pain after thoracic surgery has become one common problem, and its prevalence has been reported to reach up to 80% [31]. In this meta-analysis, we included five RCTs and 329 patients undergoing thoracic surgery. The results confirmed that pregabalin was effective to significantly reduce pain scores at 0 h, pain scores at 24 h and neuropathic pain, but demonstrated no impact on the incidence of dizziness, headache, or nausea.

Our results confirmed the efficacy of pregabalin for pain control after thoracic surgery. Although there was no significant heterogeneity for the primary outcomes, several factors may result in some bias. Firstly, the doses and methods of pregabalin were not completely same, which may cause some heterogeneity. Secondly, the thoracic surgery may include different procedures, who may cause different levels of trauma and need different surgical time. Thirdly, different patients may have various response levels to pain stimulus.

Pregabalin is a γ-aminobutyric acid analogue that binds to α2-δ subunits of the voltage-gated calcium channels in the central nervous system [32]. As a first-line treatment for neuropathic pain conditions, perioperative use of pregabalin is able to prevent the development of pain via blocking presynaptic voltage-gated calcium channels implicated in central sensitization [33, 34]. Initiating analgesic treatment before tissue damage can reduce the hyperexcitability of dorsal horn neurons and central sensitization [35].

We should consider several limitations. Firstly, our analysis was based on only five RCTs and more studies with large patient samples should be conducted to confirm these findings. Secondly, the doses, administration timing and methods of pregabalin were different in the included studies, and may cause some bias. Thirdly, different operation procedures and experience of surgeons may produce various levels of trauma and need different surgical time, which may affect the pooling results.

## Conclusion

Pregabalin may benefit to alleviate pain intensity after thoracic surgery, and more RCTs are needed to confirm this finding.

### Electronic supplementary material

Below is the link to the electronic supplementary material.

Supplementary Material 1

## Data Availability

Not applicable.
